# Beyond the three-chamber test: toward a multimodal and objective assessment of social behavior in rodents

**DOI:** 10.1186/s13229-022-00521-6

**Published:** 2022-10-25

**Authors:** Renad Jabarin, Shai Netser, Shlomo Wagner

**Affiliations:** Sagol Department of Neurobiology, Faculty of Natural Sciences, University of Haifa, Haifa, Israel

**Keywords:** Animal models, Autism spectrum disorder, Behavioral phenotyping, Emotional states, Social behavior, Social vocalizations, Three-chamber test

## Abstract

**Main:**

In recent years, substantial advances in social neuroscience have been realized, including the generation of numerous rodent models of autism spectrum disorder. Still, it can be argued that those methods currently being used to analyze animal social behavior create a bottleneck that significantly slows down progress in this field. Indeed, the bulk of research still relies on a small number of simple behavioral paradigms, the results of which are assessed without considering behavioral dynamics. Moreover, only few variables are examined in each paradigm, thus overlooking a significant portion of the complexity that characterizes social interaction between two conspecifics, subsequently hindering our understanding of the neural mechanisms governing different aspects of social behavior. We further demonstrate these constraints by discussing the most commonly used paradigm for assessing rodent social behavior, the three-chamber test. We also point to the fact that although emotions greatly influence human social behavior, we lack reliable means for assessing the emotional state of animals during social tasks. As such, we also discuss current evidence supporting the existence of pro-social emotions and emotional cognition in animal models. We further suggest that adequate social behavior analysis requires a novel multimodal approach that employs automated and simultaneous measurements of multiple behavioral and physiological variables at high temporal resolution in socially interacting animals. We accordingly describe several computerized systems and computational tools for acquiring and analyzing such measurements. Finally, we address several behavioral and physiological variables that can be used to assess socio-emotional states in animal models and thus elucidate intricacies of social behavior so as to attain deeper insight into the brain mechanisms that mediate such behaviors.

**Conclusions:**

In summary, we suggest that combining automated multimodal measurements with machine-learning algorithms will help define socio-emotional states and determine their dynamics during various types of social tasks, thus enabling a more thorough understanding of the complexity of social behavior.

## Introduction

Social behavior is a broad term that can be defined as any communication or interaction between two conspecifics of a given species [1]. Flexible and dynamic social behavior is necessary to adapt to new environments so as to ensure survival and reproductive success [2, 3]. Other than its survival-related benefits, various forms of social interactions in both humans [4, 5] and rodents [6–10] involve the activity of the mesolimbic system mediated by dopaminergic signaling (the reward system), indicating a rewarding aspect.

Social behavior encompasses many forms and can be aggressive, mutualistic, cooperative, altruistic, or parental in nature [11–13]. Social behavior entails the active and ongoing detection of cues by multiple sensory modalities and the ongoing process of reshaping the individual’s behavioral response according to the behavior of other conspecifics which comprise the perceived social environment [1, 2, 11]. More than any other aspect of cognition and behavior, social interactions of any type or nature can never be dissociated from the accompanying emotional context influencing an individual's affective state, as well as the others with whom that individual interacts [14–16]. Such characteristics make scientific explorations into the neurobiological mechanisms underlying social behavior highly challenging and have thus delayed progress in this field for many decades, relative to other fields of neuroscience [17, 18].

The study of social behavior is relevant not only for revealing the cognitive and neural processes governing its normal expression, but also for understanding how those mechanisms may malfunction to produce atypical social behavior. Abnormalities in social cue identification, impaired social skills and difficulties in maintaining social relationships are distinctive features of several psychiatric (e.g., schizophrenia), neurodevelopmental (NDD; e.g., Autism Spectrum Disorder), and neurodegenerative disorders (e.g., dementia) [19–21]. Still, the exact neural substrates and biological mechanisms underpinning abnormal social behavior in pathological conditions, which may serve as possible targets for pharmacological intervention, remain elusive [11, 22].

Given that research tools and manipulations amenable to human subjects are fairly limited, the study of the neural and molecular mechanisms underlying social deficits in various neurodevelopmental, psychiatric, and neurodegenerative diseases relies heavily on experimentation performed on animals and thus requires the generation of appropriate animal models of these pathological conditions [23, 24]. Accordingly, given their high degree of genetic similarity to humans, ease of maintenance, and rich social lives, rodents—especially rats and mice—are widely used in the research of neurodevelopmental disorders [25, 26]. The social behavior of mice and rats differs greatly, with rats showing a broader and more complex repertoire of social behaviors [25–28], and being less aggressive [29], and more rewarded by social interactions [30, 31]. Still, the larger proportion of ongoing research that considers hypotheses regarding the etiology and underlying mechanisms of NDDs is being conducted on mice, given the larger genetic toolbox available for mice, enabling the generation of mouse models with genetic alterations mimicking those found in humans [26].

Yet, despite the remarkable progress rodent models have allowed us to realize in the context of NDDs and their high construct validity, our understanding of rodent social behavior remains too limited to make any direct comparisons with that of humans. In the case of Autism Spectrum Disorder (ASD), for example, impaired altruism or lack of Theory of Mind (i.e., inferring the feelings and intentions of others) were long thought to be uniquely human traits and hence, difficult to parallel in rodents [32]. However, accumulating evidence now shows that both rats and mice have more sophisticated emotional cognition than once believed [33–35]. Moreover, while some symptoms of ASD, specifically those related to speech and linguistic communication (e.g. lack of prosody or inability to understand sarcasm) [36], cannot be mimicked by the rodent brain, given how brain substrates and mechanisms parallel to those mediating human language skills are not found in these animals [32, 36], rats and mice, nonetheless, do seem to utilize auditory cues for communication. Such cues comprise unique structures of vocalization (mainly ultrasonic) emitted in (but not exclusively) certain social contexts, which also seem to be abnormally altered in models of autism [37–39]. However, we still do not fully comprehend the exact behavioral significance of these vocalizations, nor what are the communicational deficits that their altered profiles in ASD rodent models might signify [40, 41]. It is important to note that the scope of knowledge regarding rodent social behavior is limited by the availability of tools for accessing such traits, as well as our interpretations. Therefore, behavior we deem as “less complex” may not necessarily be so, thus making the drawing of conclusions of direct links between genetic alterations and behavioral impairments even more challenging.

Nevertheless, substantial advances in the field of social neuroscience have been made in recent years with the development of cutting-edge methods for labeling, recording, and manipulating the activity of neural circuits, which have helped to reveal an increasing number of brain mechanisms and neural circuits involved in social behavior [1, 2, 14, 42–49]. In parallel, an ever-growing body of human genomic and transcriptomic studies have increased the repertoire of genes linked to social behavior in general, and specifically to disorder-associated social deficits [50, 51]. This has allowed the generation of an enormous number of genetically-modified animal lines, which may serve as animal models of pathologies associated with mutations in these genes [23, 52]. Still, the analysis of social behavior in animal models seems to be a bottleneck that significantly slows progress on this vibrant research frontier. Despite the technological achievement attained in methods that allow for testing more elaborate and inclusive paradigms, the majority of research in social neuroscience still relies on a small number of very simple behavioral set-ups [22, 24]. Moreover, these paradigms are usually employed with only one or two monitored variables, thus masking the complex dynamics of social behavior and sacrificing the ecological relevance of the collected data [29, 53–55]. While such reductionist approaches are attractive due to the high level of control over experimental conditions they provide, at the same time demanding low labor intensity, narrowing the scope of examined behaviors and over-simplification of the behavioral paradigm nonetheless represent a significant risk to translational validity. Furthermore, such strategies limit the possibility of generalizing findings made with animal models to the human brain and behavior, as well as the ability to reliably assess the potential of drugs designed to cure relevant symptoms in humans [24, 29, 32]. Increasing the number of examined variables and the complexity of behavioral assessment used for phenotypic profiling of animal models requires developing new methodologies, using detailed analysis of the dynamics of social behavior, and collecting multimodal data sets [29, 53]. This approach will help to increase the translational value of animal research into social behavior without compromising the reproducibility and accuracy of the obtained findings.

To elucidate the need for a more thorough and inclusive examination of social behavior, the three-chamber test which is considered the gold standard for assessing social behavior in both mouse and rat models, will be analyzed below as an example.

## A closer look at the three-chamber test

### What is the three-chamber test?

The three-chamber test is one of the most commonly used methods for evaluating social behavior in mouse models of ASD. It is used for assessing an animal’s preference for a social environment over a non-social environment (termed social preference or sociability) and its preference for a novel over a familiar conspecific (termed social novelty preference) [56]. In this task, the subject mouse is first placed in the medial chamber of a three-chambered apparatus for habituation. A novel same-sex conspecific placed under a wire cup serves as a “social stimulus” in one lateral chamber, while an empty wire cup located in the other lateral chamber serves as a “non-social stimulus.” Upon habituation, the walls separating the chambers are raised, allowing the subject to move freely between chambers. “Sociability” in the context of the three-chamber test is defined as the propensity of the subject to spend more time in the “social” chamber containing the conspecific, as compared to the other chamber containing the empty cup. To assess social novelty preference, a second test is carried out immediately following the first, with one chamber containing the same conspecific from the previous test, now serving as a “familiar stimulus”, and the other chamber containing a novel conspecific serving as an “unfamiliar stimulus.” “Social novelty preference”, in this context, is defined as the propensity to spend more time in the chamber containing the novel conspecific than in that chamber with the familiar conspecific [32, 57, 58]. The placement of social stimuli within wired cups prevents these mice from freely moving in the arena and restricts their direct physical contact with the subject. This, in turn, attenuates the expression of aggressive and sexual behavior while still allowing the subject to explore and detect sensory cues (namely, smell, sight, sound, and touch). Under these conditions, the subject mouse is solely in control of actively seeking and investigating the social stimulus [53, 57].

The three-chamber test thus provides an elegant but simple design with high experimental control and offers easy objective scoring, as compared to a social interaction test involving two freely moving animals [53, 59]. Indeed, the three-chamber test represented a breakthrough in the field of social neuroscience and now serves as a fundamental instrument that offers a wide range of applications. These include phenotyping social deficits in transgenic and environmental mouse models of ASD [60–66], investigation of social deficits during development [8, 67, 68], comparing distinct strains and genotypes [56, 58, 59, 69, 70], as well as testing the effects of pharmacological treatments and other manipulations on social behavior [38, 63, 71–74].

### Limitations of the three-chamber test

Despite its tempting simplicity and high degree of experimental control, the three-chamber test suffers from multiple limitations. These are discussed below.

#### Limited number of monitored variables

The assessment of social behavior by the three-chamber test is usually restricted to monitoring one or two variables, usually the time spent in each chamber and/or the time spent in proximity to distinct wire cups. However, evaluating social behavior using only one or two variables reduces its complexity to a single dimension. Moreover, estimating sociability by “time spent in chamber” may not be a very reliable reflection of social propensity, given that time spent in a given chamber does not necessitate an active and direct investigation of the stimulus in that chamber. Furthermore, social interactions are reflected by multiple behavioral variables that are in constant interplay and dynamically change over time. In the context of the three-chamber test, such variables include the number and length of individual bouts of interaction with the stimuli, the rate of transition between stimuli, the progression of stimulus investigation over the duration of the test, and the periods spent by the subject in non-social activities, such as grooming and resting. Modeling social behavior in the social preference test with several of these variables revealed two distinct phases of subject mouse behavior during the test, namely, an “exploratory phase” in which the subject’s investigation is merely driven by curiosity and exploration, and the “interaction phase”, when the subject begins to show an increased tendency for interaction with the stimuli [75]. Therefore, future assessments of rodent social behavior should incorporate more nuanced variables, such as accurate detection of body posture, combined with an analysis of behavioral dynamics during the paradigm being employed. Such an approach is not only crucial for enhancing the translational validity of behavioral testing, it is also important for differentiating between distinct aspects of social behavior. Such aspects may be mediated by distinct molecular and neuronal mechanisms that cannot be identified solely by relying on the “time spent in chamber” variable [53].

#### Non-standardized protocols

Despite the straightforward approach of the three-chamber test, there is still a lack of consensus regarding a standardized protocol for its use. Variations in protocols include differences in the habituation period, currently ranging from 5 min [57, 58, 60, 69, 74, 76] to 10 min [32, 38, 59, 77–82], and even 20 min [63, 83, 84]. Protocols also differ in terms of what constitutes a “non-social” stimulus. While some protocols introduce a novel object placed under a wired cup in the non-social compartment [38, 68, 77, 78, 85], others place a wire cup with nothing underneath [32, 57, 59, 60, 63, 69, 76, 80–84]. Another variation includes the portion of the arena to which the subject animal has access during the habituation phase; some protocols limit the habituation space to the central chamber of the three-chamber apparatus [32, 57, 58, 60, 74, 80], while others allow the animal to explore the entire arena [38, 69, 76–78, 81, 82]. Given that the main variable used to estimate sociability is time spent in each chamber during the testing phase, pre-test habituation to the central chamber alone introduces confounding variables irrelevant to social tendency, such as spatial preference, anxiety, and novelty-seeking that may drive the animal to spend more time in one chamber over the other. CD1 outbred mice, for example, failed to exhibit social preference when they were habituated to the central chamber only. Rather, they showed intact social preference when exposed to all three chambers during the habituation phase [79].

The variety of testing methods in use has also contributed to discrepancies in the phenotyping of several ASD mouse models, with varying conclusions regarding levels of sociability, including *Shank2*-KO [81, 82],*Cntnap2*-KO [63, 77, 83, 86], and *16p11.2*^+/−^ mice [87, 88]. In one attempt to examine the effect of this procedural variability on detected deficits in social behavior, Rein et al. [56] tested two versions of the three-chamber test. One version included a pre-test phase in which subjects were introduced to two identical objects within the cups so as to familiarize them with objects being presented inside the cups. This method was designed to minimize variability caused by novelty-driven interactions with the cup and to prevent “muddying” of detected preferences for the social versus the non-social stimulus. The other tested version compared the subject’s interaction with a social stimulus placed in one chamber versus an empty cup serving as the “non-social” stimulus in the other chamber. The two tested versions yielded different sensitivities in detecting social preference deficits in multiple ASD mouse lines, including *Shank3*^+/ΔC^, *Cul3*^f/−^, and *16p11.2*^+/−^, thus demonstrating that protocols involving an inanimated object as a non-social stimulus rather than an empty cup are more sensitive to social preference deficits in ASD models [56]. Taken together, such findings highlight the need for standardized protocols that allow for consistent phenotyping of ASD models, and for the use of more reliable measurements to estimate social behaviors that can persistently and accurately capture changes in social behavior while remaining insensitive to variations in the protocol used.

#### The need for multiple tests

Even if social deficits are detected using the three-chamber tests (or any other social task for that matter), one test alone cannot provide the sole basis for reaching the conclusion that a certain mouse model is socially impaired or not. The *16p11.2*^+/−^ line used to model ASD, as an instance, displayed intact social preference in multiple studies [83, 84, 88], yet exhibited deficits in other behavioral tasks, including sex preference and emission of mating calls [39, 88], social recognition memory and habituation [83, 84], and the degree of direct social interaction [87]. Another example is the *Iqsec2* A350V line, which showed intact social preference and social novelty preference in the three-chamber test, yet also displayed deficits in sex and emotional state preference [73].

The need to employ multiple behavioral assays for the phenotyping and exploration of NDD animal models was recently highlighted in a review by Silverman et al. [27]. ASD, for instance, is a heterogeneous disorder with high percentages of comorbidity and multiple associated symptoms occurring in subsets of autistic individuals, such as seizures, anxiety, mental retardation, hyper- or hypo-reactivity to sensory stimulation, and motor abnormalities [36, 80]. Therefore, associated symptoms are a major source of potential artifacts that can confound the interpretation of the phenotype revealed in a mouse model. For example, a mouse model for ASD might exhibit an abnormal social approach and social recognition due to innate high anxiety levels, impaired sensory perception, or motor defects and not necessarily due to altered social motivation. Thus, phenotyping of mouse models for NDDs should not be limited to “single tasks” that examine core features of the disorder, such as lack of sociability or repetitive behavior, but rather should also include a battery of tests that address associated symptoms like anxiety, sensory functioning, and motor fitness, which may offer alternative explanations for behavioral abnormalities [27].

#### Confounding variables

In addition to potentially being influenced by spatial-related processes, as mentioned earlier, the social behavior of a tested subject may be affected by other confounding variables that can bias or mask the results of any social task. Such factors include social rank and aggressiveness [22, 53, 89], strain [58, 59, 69], experimental settings, such as lighting conditions and the use of a novel testing arena [52, 59, 89], housing conditions, including the size, genotype, and gender composition of the litter [56, 90], and even individual differences in temperament and personality [91, 92].

It is important to note that the limitations discussed above are not necessarily exclusive to the three-chamber test and can be considered as relevant to a wide variety of gold standard tests in other fields. For example, two of the most common tests for assessing anxiety levels and anxiety-related behaviors in both mice and rats are the Elevated Plus Maze (EPM) and the Open Field (OF) tests, both of which rely on the innate conflict of rodents between the drive to explore a new space and the fear of open spaces [93]. The EPM consists of a plus-shaped maze with two open arms and two closed arms inter-connected by a central platform elevated above the ground. Anxiety is measured by calculating the percentage of time subject animals spend in the open versus the closed arms, with more time spent in the closed arms indicating higher anxiety levels. In the OF test, the subject animal is placed in a box-shaped arena with walls and the animal's trajectory is tracked during the session. Anxiety is then inferred by calculating the percentage of time spent along the walls of the arena versus the center, with more time spent along the walls indicating higher levels of anxiety [25, 93]. While these tests have high ecological value, with an economic and straightforward design, and show sensitivity to anxiogenic and anxiolytic pharmacological treatments [94], they still suffer from multiple caveats similar to those of the three-chamber test. For example, both tests, like the three-chamber tests, have no single, agreed-upon standardized protocol adopted across laboratories [94, 95]. Performances in these tests were also shown to be influenced by multiple confounding variables, including species, strain, gender, age, housing conditions, prior handling and exposure to stress, illumination levels, and prior test experience [93–95]. Furthermore, the behavior of tested subjects in the EPM was shown to differ on a minute-to-minute basis [94], indicating the redundancy of estimating anxiety by calculating one or two variables, like the number of entries or total time spent in open/closed arm. Lastly, it was found that reliance on solely one test, like the EPM or OF test, is not reliable for determining anxiety levels in rats, given that performance in one test was not correlated with performances in other tests for anxiety. This again stresses the need for profiling anxiety by applying multiple tests [96].

#### Affective states

Finally, currently used behavioral paradigms for phenotyping social deficits in ASD models often overlook a key component of any form of social behavior, namely, the emotional state of the subjects involved. Accumulating recent evidence shows that rodents possess higher levels of emotional cognition than once believed, and that their emotional state can affect their behavior by generating cognitive biases and inducing differential effects in response to the same stimuli [97, 98]. Despite their robust influence on behavior, assessing emotional states in animal models still relies on a fairly limited number of tools, thus creating a gap that prevents any reliable comparisons of deficits in social behavior exhibited by human patients and social deficits characterized in animal models. Validated measures of an affective state can help in developing improved models and treatment options for human emotional disorders and may provide additional information regarding the neural mechanisms behind such complex forms of behavior [97]. The following chapter will discuss this requirement and offer possible solutions.

## Socio-emotional states in rodents

Although there is no consensual definition of emotions, these can be viewed as central states triggered by intrinsic or extrinsic stimuli processed in particular neural circuits and which drive behavioral, cognitive, somatic, and physiological responses [99]. Such a definition of emotions does not necessitate the existence of subjective consciousness as a requirement for experiencing emotions. Therefore, basic and more primitive forms of emotional states can be found in animals [99–101]. This comes as no surprise, since research in rodents has already established the existence of negative emotional states, like fear and stress [102], that elicit distinct behavioral and physiological changes spanning multiple modalities [100, 101]. Emotional states in rodents can be recognized as complex and flexible reactions to environmental events that can persist for some time, influencing other aspects of cognition, and affecting subsequent behavioral decisions [99, 101]. Rats’ emotional states, for example, were found to influence their decision-making behavior, as seen in the Ambiguous-Cue Interpretation Test, in which animals are first trained to associate one cue with a rewarding outcome and a second cue with avoiding punishment or a less rewarding outcome. Animals experiencing a negative affective state were found to exhibit a “pessimist” judgment of an ambiguous cue and respond to it as if it predicted the negative/less rewarding outcome, while animals experiencing a positive affective state displayed an “optimist” cognitive bias that led them to deem the ambiguous cue as being predictive of a rewarding outcome [103, 104].

**Fig. 1:**
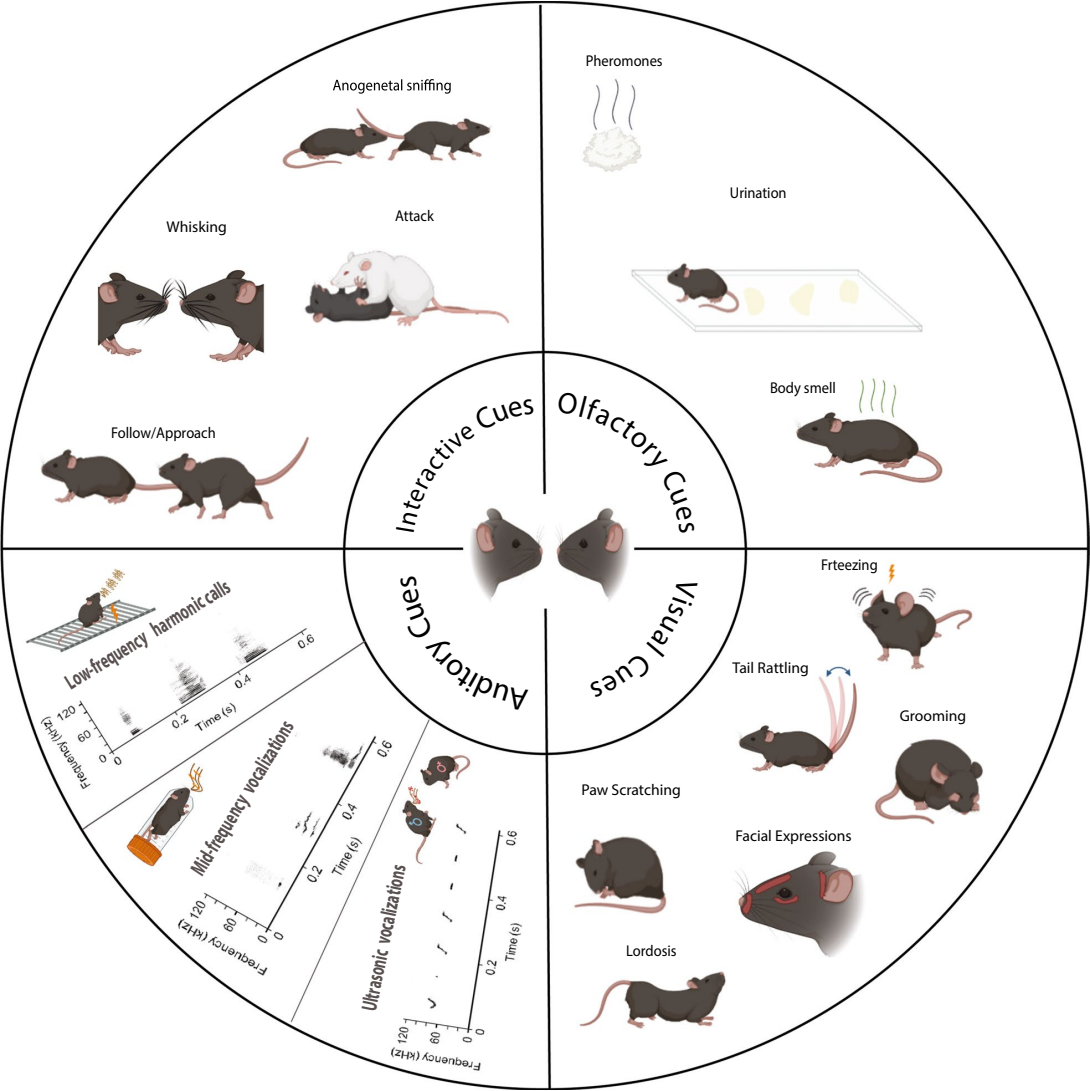
Modalities of social communication in mice. Socio-emotional interactions are mediated by cues of multiple modalities portrayed in the figure. These modalities include interactive cues between two (or more) conspecifics, such as anogenital sniffing, whisking, attack, and approach, olfactory cues, like body odor, pheromones, and urinary scent-marking, vocalization of low-, mid- and ultrasonic frequencies emitted in different contexts and visual cues displayed by another conspecific, including like facial expressions, tail rattling, freezing, paw scratching, grooming, and lordosis

Moreover, both mice and rats communicate emotional content using multiple modalities, as shown in Fig. 1 for mice. These include postures like freezing, vocalizations of varying frequencies [41, 105, 106], scent marking and release of pheromonal cues for communicating social or sexual status, as well as affective states [107–109], and distinct facial expressions in response to emotionally salient events [110]. Thus, the emotional state experienced by a mouse or rat can influence its social behavior toward a fellow conspecific. Evidence supporting the expression of complex forms of socio-emotional behavior, like pro-social and empathic behaviors, in rodents has recently begun to accumulate [91]. Prairie voles were shown to engage in higher levels of allogrooming of their partner when re-united after a 24 min separation if their partner received an electric shock during the period of separation [34]. Exposure of rats to a stressed and fear-conditioned cage-mate increased allogrooming of that cage-mate, facilitated the acquisition of avoidance behavior in the training phase of a fear conditioning paradigm, and increased fear memory retention [16, 19]. Rats were also shown to modify their behavior and refrain from pressing a food-delivering lever that also delivers a foot shock to a cage-mate [111, 112]. Moreover, rats introduced to a trapped cage-mate quickly and consistently freed their cage-mate, and when given a choice between pressing a lever to obtain chocolate or pressing a lever to release a trapped cage-mate, the rats preferred to free the trapped cage-mate [113]. This willingness to cooperate with other conspecifics in rats was influenced by the value of previous benefits received by those conspecifics- female Norway rats were more willing to provide cereal flakes to a partner who previously provided them with a piece of banana than a partner who had earlier provided them with a carrot [114]. However, rats still exhibited pro-social behavior and chose to provide food rewards to a cage-mate even without any direct self-benefit resulting from their choice [115].

In mice, evidence for such complex forms of pro-social behavior is more scarce. Still, available findings indicate that mice possess the ability to recognize and respond to the distress of other conspecifics. For instance, exposure to a cage-mate in pain increased pain behaviors in observer mice also experiencing pain and induced hyper-mechanical and -thermal sensitivity to nociceptive stimulation [35, 116]. Moreover, mice exposed to a shocked cage-mate displayed increased social approach and allogrooming toward the stressed mouse, indicative of an emotional response [117].

While the existence of high-level social cognition that can mediate abilities, such as empathy and Theory of Mind, in rodents is still under debate, it is well-accepted that rodents do show emotional contagion, as evident in tasks such as social transfer of fear, pain, and food preference [14, 35, 118, 119]. In emotional contagion, the subject’s attention to the “state” of another automatically activates the same state in the observer, thus increasing the probability of behavior driven by that emotion and allowing for rapid adaptation to environmental challenges [15, 29]. An essential component of emotional contagion is the ability to detect, recognize, and react to the emotional state or arousal of other conspecifics. To assess affective state discrimination ability in rodents, a behavioral paradigm was recently developed by Scheggia et al*.* [120], in which emotional state recognition (also termed ‘affective state discrimination’) was estimated by comparing the time an “observer” subject spent investigating a “demonstrator” in a neutral state versus one under an arousing affective state (positive or negative). C57BL/6J mice of both sexes preferred the emotionally aroused conspecific experiencing either a positive or a negative affective state over a neutral conspecific. This ability depends on oxytocin signaling in the paraventricular nucleus (PVN)-Central Amygdala (CeA) pathway, and on inhibition mediated by somatostatin-expressing (SOM) interneurons in the pre-frontal cortex (PFC) [20, 120]. These behavioral observations imply that demonstrators transmit cues about their affective state, which are then detected by observers using different sensory modalities. These sensory cues may eventually converge on a common neural circuit dedicated to processing emotional cues, encompassing areas like the PVN, CeA, insular cortex, and PFC [118]. Paradigms such as these may be used to characterize deficits in affective state discrimination in animal models of human pathological conditions. This type of examination seems to be especially relevant to ASD, a condition in which disruption of emotional cognition and Theory of Mind-related processes is a core feature [119, 121]. Such abilities can be independently hindered regardless of the general social propensity of the tested subjects. *Iqsec2* A350V-mutated mice, for example, exhibit deficits in specific social interactions that include emotionally-arousing stimuli [73]. Therefore, the application of behavioral tests designed for assessing emotion-cognition-related processes might yield greater insight into behavioral deficits related to ASD.

The proper assessment of emotional states in animals will require multi-dimensional methods aimed at defining complex signatures that reflect a subject’s emotional state and able to capture how this state affects social behavior. One such approach is discussed below.

## A multidimensional approach for phenotyping social behavior

Socio-emotional states involve various neuronal, hormonal, physiological, and behavioral processes that interplay to enhance the survival and success of an individual in any social context [65, 99, 101, 122]. Fearful situations in humans, for example, elicit a wide range of physiological (e.g. increase in heart rate, blood pressure, respiration, and sweating), hormonal (e.g. HPA-axis activation, secretion of cortisol, and increased adrenaline levels), and behavioral changes (e.g. facial expressions, body posture, and freezing or fleeing) [100, 123, 124]. Therefore, to identify socio-emotional states in an ethologically valid manner and distinguish between them, one can rely on the complex signatures of such states across different modalities. This approach requires capturing and correlating as many aspects of a subject's behavior and physiology as possible. Such multimodal analysis provides a detailed and wide perspective that is expected to be much more informative than are commonly used measures of “time spent in chamber”. Moreover, subtle differences in specific behaviors or physiological variables may reflect unique emotional states of a subject or its responses to the affective states of other conspecifics. Such findings may also help to delineate differences in the neural circuitry responsible for varying behavioral responses during distinct types of social interactions [98, 118].

In the following section, we will detail several “fronts”, including some behavioral and physiological variables of different modalities, that have been shown to be involved in social behavior. The section will also describe the latest methodological advances for measuring and analyzing these variables and discuss how such variables may serve as good candidates for providing meaningful information on complex aspects of social interaction, like the socio-emotional state of the subject animal.

### Systems for automated behavioral analysis

Social interactions usually involve multiple individuals displaying a dynamic and high-dimensional repertoire of behaviors influenced by their own motivational and emotional states, as well as those of their partners [3, 125, 126]. As such, accurate and thorough quantification of social behavior is needed to understand the exact neural basis mediating its intricacies [127, 128]. While human analysis and manual annotation have their benefits, like the ability to differentiate between closely similar behaviors that can otherwise be prone to faulty classification by automated analysis methods, manual analysis of social behavior still has its downfalls. Besides being time-consuming and tedious, human analysis is limited by the observer’s ability to visually perceive and follow complex sequential behavior, making it prone to human error, bias, and variable inconsistencies [2, 125]. Accordingly, numerous attempts to develop objective computerized tracking systems to analyze animal behavior, a task that has proven to be highly complex, have been made. Table 1 lists some presently available tracking, pose-estimation, and classification computerized tools for rodent behavioral analysis.

**Table 1:** Available tracking, pose-estimation, and classification programs for rodent behavioral analysis

Name	Function	Number of subjects	General description and relevant features	Measured variables	Citation
UMA tracker	Tracking	Multiple subjects (without identity preservation)	An image-based tracking algorithm Allows the application of multiple image processing algorithms so as to choose the most suitable An option for manual correction of tracking and trajectory swapping errors Requires arenas with high contrast	Trajectory, interaction times in regions of interest (ROI)	[132]
Rodent arena tracker (RAT)	Tracking	Individual subjects	Machine-vision tracking device that is inexpensive, has low battery demand and does not require a tethered computer Requires a high-contrast arena Real-time online image processing Can be synchronized with other devices for pellet dispensing\optogenetic stimulation, etc.	Trajectory and speed	[133]
Mousemove	Tracking	Individual subjects	A software for centroid-based tracking; thus does not offer orientation-dependent information Requires high-contrast circular arenas Restricted to video resolution of 320 × 240 [151] Batch processing option [129]	Trajectory, traveled distance, speed, turning and curvature in the entire arena or within a ROI	[134]
Mouse tracking	Tracking	Individual subjects	Neural network-based tracker for long periods (days) in multiple, complex, and dynamic environments The option to train a new network suitable for the user’s need with minimal training data needed (minimum of 2500 annotated images) Indifferent to coat color or animal size	Traveled distance, speed	[126]
Automated rodent tracker (ART)	Tracking	Individual subjects	A rule-based system for tracking a rodent’s nose and body points with minimal user interference needed Detection of orientation and head-direction of subjects Requires high-contrast arenas Option for batch processing of multiple videos	The frequency of certain behaviors (exploration, moving forward, turning, interacting with a ROI), locomotion variables (speed, distance), and body size estimation	[135]
Janelia automatic animal behavior annotator (JAABA)	Behavior Classification	Single or multiple subjects	Machine learning algorithm for neural networks-based behavioral classification using animal trajectory Users annotate a small set of video frame to create classifiers for detecting behaviors of interest in screen-scale data sets Can operate on the output of multiple tracking systems (e.g., Ctrax, MoTr)		[127]
MoTr	Tracking	Multiple subjects	A software for long-duration tracking (days) in home cage environment, with identity preservation Identity is assigned by coloring subjects with distinct bleach patterns that are detected and learned by the tracking software Detects the position and orientation of the animal based on previous frames by applying an Expectation–Maximization algorithm Suitable for quantification of social behaviors with long-scale dynamics (dominance, aggression, courtship)	Preferred location, preferred associates, following rate and duration, speed	[142]
DeepLabCut	Pose estimation		Based on transfer learning of deep neural networks with minimal training data needed [193] for classifier creation Can be used for detecting the pose, orientation, and posture change of body parts of multiple free-interacting mice Option for retraining the network for fine-tuning to a specific need/task by providing it with labeled data on annotated body parts locations	Trajectories, traveled distance, and location of annotated points	[149]
MiceProfiler	Tracking	Two subjects	The software requires no specific tagging of subject animals by implementing a physics engine capable of maintaining identity even after occlusions and with hidden body parts Can detect the orientation of the mouse’s head (oral-oral, oral-genital, side-by-side interactions) The system is limited by its need for supervision and correction by an expert [143]	The frequency, duration, type (follow, escape, investigation), and temporal evolution of pre-determined behavioral events The identity of the animal initiating an action (follower/leader), and the response of the other conspecific	[125]
A 3D-video-based computerized analysis of social and sexual interactions in rats	Tracking	Two subjects	The system is used to detect behavioral events that include vertical changes in posture (rearing, mounting) by using four depth cameras positioned at different viewpoints to extract a 3D image of two freely-moving rats. The merged extracted image is fitted into a “skeleton” model by physics-based algorithm to estimate the location of four body parts (head, neck, trunk, hip) for identifying spatio-temporal patterns of these parts May need manual corrections for identity swaps and dislocated body-parts	Frequency, latency, and duration of dynamic behavioral events like rearing, head-head\hip contact, approach, follow and mount	[144]
RFID-assisted socialscan	Tracking	Multiple subjects	A system for long-term tracking (days) tracking in ethologically relevant environments, and with identity preservation Identity preservation is obtained through radio frequency identification – each subject is implanted with a RFID chip that transmits a unique radiofrequency detected by RFID antennas placed underneath the arena that is then synchronized with the video frames for identity assignment An option for adjusting\adding new parameters for detection by the user Animal identity is preserved even when out of frame, thus enabling the attachment of other components to the arena (nests and enrichments)	Detection of specific social events (identified by built-in rules), like approach, contact, follow, leave, and locomotor activity within ROIs	[141]
Autotyping	Tracking	Individual subjects	A toolbox for locating and measuring time spent in ROIs in multiple behavioral tasks, including open field, fear conditioning, elevated zero maze, Y\T-maze, spatial\novel object recognition, and three-chamber task Requires high-contrast arenas	Interaction time\time spent in a given location, number of exploratory bouts, approach angle during bouts of interaction, distance traveled	[137]
ToxTrac and ToxId	Tracking	Multiple subjects	An open-source software for image-based tracking, with high processing speed (less than 25 frames per second), integrated distortion correction and camera calibration, and identity preservation of multiple subjects Can be used in multiple arenas ToxId algorithm can be implemented in ToxTrac and enables identity preservation of multiple “untagged” animals by linking trajectory segments using their intensity and Hu-moments with no training or complex configuration steps or access to past and future frames needed	Average speed, acceleration, distance traveled, time spent near\in a ROI	[140, 151]
Mouse action recognition system (MARS)	Pose estimation and behavior classification	Two subjects	Automated pipeline and software tools (deep learning based) for supervised training and evaluating of novel pose estimation, behavior classification, and joint visualization of neural and behavioral data Subjects need to have different coat colors (one black, one white) Include three pre-trained supervised classifiers trained to detect attack, mounting, and close investigation events An option for training MARS pose and behavior models for creating user-specific classifiers from manually annotated videos (minimum of 1500 annotated frames needed for training) Suitable for head-mounted animals with implantations Includes an open-source interface Behavior Ensemble and Neural Trajectory Observatory (BENTO) for synchronous display, navigation, and analysis of behavior annotations, audio recordings, and recorded neural activity	Frequency and time spent in specific behavioral events (attack, mounting, close investigation) Can detect orientation-sensitive behaviors like face\anogenital- directed sniffing)	[128]
TrackRodent	Tracking	Up to two subjects (without identity preservation)	Suitable for rats and mice Requires high-contrast arenas Suitable for implanted animals Options for multiple tracking algorithms based on species (mouse\rat), coat color, head-\body-based tracking, and head implantation	Total time of investigation of ROI, bouts of investigation and their distribution, transitions between defined regions, investigation along time, and intervals between investigation bouts	[75]
Idtracker.ai	Tracking	Multiple subjects	An algorithm and software for extracting the trajectories of freely-moving and unmarked animals in high-contrast arenas The software is based on two convolutional networks, one for detecting events of animals touching or colliding, and one for assigning identification to the detected animal using classification analysis The software can be used for detecting multiple subjects of various species in different environments but requires large training data to adapt to new animals and experimental settings Idtracker’s ability to maintain the identity of multiple mice in a long recording given their deformable geometric shape is yet to be established [143] High computational demands [131]	Trajectories of detected animals	[148]
Simple behavioral analysis-SimBA	Behavior classification	Two subjects	An open-source software that uses pose-estimation to create supervised machine learning predictive classifiers of rodent social behavior Requires different coat coloring Uses labelling interfaces and pose-estimation data from DeepLabCut and DeepPoseKit to annotate body parts on subject animals	Durations and frequencies of classified behaviors	[150]
Video-RFID tracking system	Tracking	Multiple subjects	A system for automated location tracking within a semi-naturalistic setting, and for long periods of time (from minutes to several weeks) Identity preservation is obtained through radio frequency identification—each subject is implanted with a RFID chip that transmits a unique radiofrequency detected by RFID antennas that is then synchronized with the video frames for identity assignment The system integrates MiceProfiler algorithms for improving identity detection and defining mouse-body orientation for more sensitive behavioral characterization	Locomotion (travelled distance and time spent in running, walking, hiding, sleeping, and staying still), and the number of social events (include avoiding, being-avoided, chasing, being-chased) that can be later used for quantifying social dominance	[147]
Live mouse tracker	Tracking and behavior classification	Multiple subjects	A real-time tracking software combining computer vision, machine learning, and RFID identification methods for tracking and classifying behavior in a home-like environment for up to several days The tracking is possible with any coat color, wired animals, and enriched environments Identity of subject mice is appointed using RFID and machine learning algorithms with the ability for the user to monitor the quality of the tracking live during the experiment the setup includes a depth camera that enables the extraction of animals’ orientation (head–tail) and the detection of various head parts, like ears and nose The option to synchronize behavioral tracking with USV recording through the LMT USV Toolbox thus enabling the investigation of spontaneously emitted USVs in home-like environments. The system does not record audio continuously, but only when a certain power threshold is crossed, and then uses machine-learning-based classifier to filter out recorded files of noise. Extracted USVs are then correlated with behavioral events detected by LMT. Cannot appoint the identity of the emitter	Based on changes in shape geometry, the system is able to detect and classify up to 35 different behavioral events related to individual behavior, social dyadic events, dynamic events (escape and follow), and subgroup configuration events	[143]

One core requirement of any tracking system is the ability to locate an animal's position and separate it from its surroundings [129]. To locate an animal within a given frame, some tracking systems employ computer vision algorithms for background subtraction followed by segmentation, techniques that usually require simplified and fixed arenas with a high level of contrast between the background and the target for adequate separation [2, 129–131]. Such demands require behavioral testing to be constrained to specific simplified arenas, which may compromise the translational validity of the testing environment, increase the anxiety levels of the tested animals [89], and limit the strain repertoire of possible subjects, depending on their coat coloring [132–137]. In addition, some tracking systems track the position of an animal by locating its center of mass (centroid) and reducing the tracked animal to a single point, thereby providing information limited to the subject’s location, with no information regarding the orientation and/or directionality of behavior [131, 133, 134, 138]. Neglecting directionality in behavioral analysis overlooks a rich source of valuable information in the context of social behavior, given that some social behaviors require the identification of the animal’s orientation. In rats, for example, anogenital sniffing is considered an affiliative action of social investigation, while face-to-face investigation might increase the probability of attacking a subordinate rat [139]. Another example comes from the work of Hong et al. [2] in which pose estimation of freely interacting mice revealed a significant reduction in the time spent in short (< 4 cm) head-body distances by BTBR subjects investigating a BALB\c stimulus, as compared to C57BL/6J subjects.

Furthermore, while some tracking systems are adequate for tracking one animal [133–138], free social interactions involving two animals at the least introduce the challenge of tracking multiple subjects and maintaining their identities over the course of analysis, including periods of close physical proximity (huddling) or following occlusions [129, 140]. Possible solutions to this issue include various forms of “artificial marking” of tested animals, whether by coloring an animal with distinct dye patterns or implanting the animals with RFID chips that emit radio frequency signals unique to each subject [141–143]. Another innovative solution for the identity preservation issue is the use of multiple depth cameras covering multiple viewpoints for 3D depth filming of social interactions [144].

**Fig. 2:**
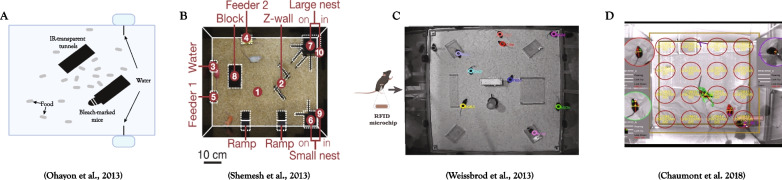
Systems for tracking group behavior of mice in a complex environment. Tracking systems in a semi-naturalistic environment for long-term tracking of multiple individuals within a group context, including the systems described in **A** [142], **B** [146], **C** [147], and **D** [143]. The picture in subfigure C was provided by the authors of the paper [147] for the purpose of this review

Another aspect of social behavior that presents a challenge for computerized behavior analysis is group dynamics. While most social tests focus on dyadic interactions between two mice [36, 145], the behavior of animals in a group cannot be predicted by models based solely on the behavior of the individual or the behavior of pairs, indicating that social behavior is determined by relatively complex interactions that include more than one other animal [146]. For capturing group behavioral dynamics, some tracking systems have the ability to track animal activity across days in a semi-natural habitat while maintaining identities (see Fig. 2 for several examples), offering a relevant tool for investigating social behavior with long-scale progressions, such as dominance, sexual courting, and the identification of persistent personality traits [126, 142, 143, 146, 147].

Advances in machine learning approaches and neural network training have led to the development of tracking algorithms capable of detecting multiple untagged and freely moving animals while maintaining their identities. One example is idtracker.ai [148], an algorithm and software that implements two convolutional networks, one for detecting collision events between subjects and one for assigning identities to the detected animals using classification analysis with no need for any artificial “tagging.” Deep learning approaches also enabled the development of pose-estimation systems for detecting and tracking changes in the postures and positions of user-defined body parts, allowing a closer look at fine motor changes involved in the performance of certain behaviors [131, 149]. A leading example for a deep-learning based pose-estimator is DeepLabCut, which employs transfer learning of neural networks to simultaneously estimate the body positions of multiple animals. DeepLabCut is a deep convolutional network pre-trained for object recognition on images from the ImageNet dataset. Due to transfer learning, the network needs minimal training data of manually labeled and annotated frames to fine-tune weights within the network to detect and classify events relevant to the specific needs of the user [149].

Machine learning-based approaches also contributed to programs like JAABA [127] and SimBA [150], used to classify behavior through supervised learning into user-specified categories by training neural networks with manually annotated data. Such programs can be beneficial in the context of social behavior and for quantification of distinct behavioral events like grooming, attacking, and mounting that might otherwise by missed by conventional top-view 2D position-based tracking.

Thus, developing software with features including animal tracking, pose estimation, and machine-learning-based options for supervised behavioral classification will provide high-resolution insight into the nuances of social behavior and facilitate the study of their underlying neural circuits and genes. It is noteworthy that most available tracking systems are better suited for tracking target animals in simple postures. Therefore, the identification and interpretation of a subject’s affective state and the meaning of each displayed change in behavior or movement may be hard to achieve relying on vision-based tracking alone.

### Vocalizations

Vocalizations emitted by rodents serve as a communicational tool that varies in frequency range according to the emotional context [90, 105, 152, 153]. Pups elicit 30–60 kHz calls with varying acoustic features when separated from the dam, resulting in approach and retrieval behaviors and reducing attack and rough handling by the dam [105]. Since pup calls emission is modulated by maternal cues and affected by anxiolytic/anxiogenic drugs, ultrasonic vocalization (USV) analysis in pups can be considered a suitable model for studying the development of emotionality in rodents [90, 105].

**Fig. 3:**
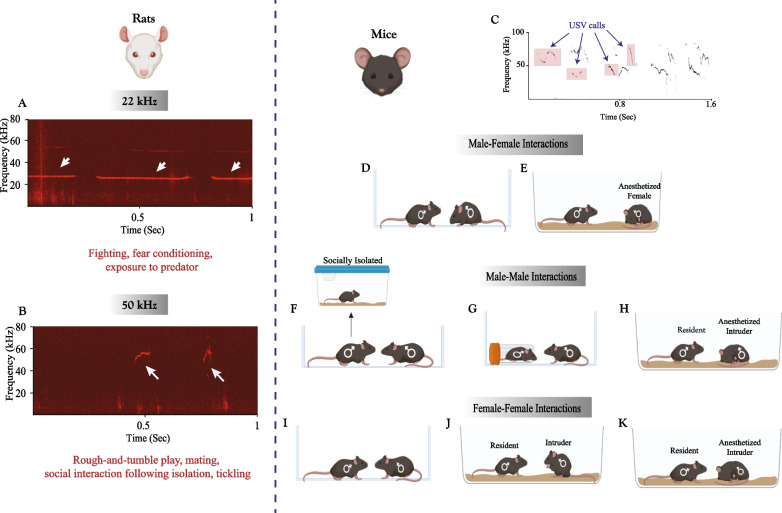
Social contexts of ultrasonic vocalizations in adult rats and mice. USVs emitted by adult rats can be divided into two categories: **A** 22 kHz USVs emitted in aversive contexts and **B** 50 kHz USVs emitted in appetitive contexts. **C** An example of various USV elements emitted by male mice during a male–female interaction. Social contexts of USV emission by mice include male–female interactions **D** when both animals are awake or when **E** the female is anesthetized, and male-male interactions in which the vocalizer **F** has been socially isolated prior to the encounter, **G** held in a restrainer, or **H** introduced to an anesthetized intruder. In female-female interactions, USVs are emitted **I** in response to a novel female, **J** in response to an awake female intruder, and **K** in response to an anesthetized female intruder

USVs were also shown to convey emotional content in adult rats [105] (Fig. 3A, B). These animals emit USVs at a 22 kHz frequency in negative emotional contexts like exposure to predators, threats, pain, and during withdrawal from drugs, such as benzodiazepines and psycho-stimulants. In contrast, 50 kHz USVs are emitted in more affiliative contexts, including play solicitation, sexual interactions, social exploration, and drug-induced reward states [10, 29, 91, 105, 152, 154]. The emission of 22 kHz alarm calls was found to elicit freezing behavior in ‘listeners’ who had previous experience with the aversive stimulus used to elicit the calls, accompanied by increased activity of brain regions regulating fear and anxiety, including the amygdala, periaqueductal gray (PAG), and hypothalamus [10, 155]. The emission of 50 kHz USVs, on the other hand, was found to encourage social approach and cooperative behavior and to establish and maintain social contact. These events were accompanied by decreased activation of the amygdala and increased activation of brain regions implicated in reward, like the nucleus accumbens, mediated by increased dopaminergic signaling in the ventral tegmental area (VTA) [10, 152, 153]. Both the 22 and 50 kHz USVs were affected by social experience, with prolonged social isolation decreasing the emission of the 22 kHz call and increasing the emission of 50 kHz calls during play interaction and in anticipation of tickling, indicating an increase in social motivation [10, 152].

Mice were also found to emit a variety of vocalizations spanning a wide range of frequencies that can be divided into non-USVs, comprisng low-frequency harmonic calls (LFHs) and mid-frequency vocalizations (MFVs), and USVs. LFHs, or “squeaks”, are composed of harmonic complexes with a power audible to humans and frequencies below 5 kHz. These vocalizations are emitted mainly in aversive contexts, like pain, agitation, and fighting [106]. MFVs are a non-USV category identified by Grimsley et al. [41], encompassing vocalizations with a frequency range between 9 and 15 kHz. They are emitted under different types of restraint, depicting a negative emotional state of the emitter and eliciting stress and anxiety in the listener [106] (see Fig. 1). However, mice predominantly emit USVs in social contexts with frequencies above 20 kHz. Mice USVs present many structurally and temporally complex acoustic features that can vary across developmental stage [10, 90, 105, 156], genetic strains [157, 158], gender [159–162], and social context [163–165] (Fig. 3C). Mice USVs can be classified into different syllables, defined as units of sound composed of one or more tones and separated by silent pauses [166–168]. Syllables can be categorized based on their acoustic variables, i.e., bandwidth, duration, amplitude, and shape [158]. Still, there is no universally accepted classification for mice USV syllables [158, 167–171]. To capture and analyze USVs in rodents at different levels of complexity, multiple tools have been developed for USV detection and classification, some of which are summarized in Table 2. It is important to note that while some attempts to understand the role of the different ultrasonic vocalizations in mediating behavior [169, 172, 173] and the context in which they are produced [164, 170, 174] have been made, the functional extent and exact meanings of the varying variables of mouse USVs in social interactions remain unknown [171].

**Table 2:** Available programs used for USV analysis in rodents

Name	General description and relevant features	References
WAV-file Automated Analysis of Vocalizations Environment Specific (WAAVES)	An automated USV assessment program utilizing MATLAB’s Signal and Image Processing Toolboxes and customizes filters to separate USV calls from noise and assign each USV into one of two categories: 50–55 kHz and 22–28 kHz USVs Appropriate for rat call analysis Different test environments (e.g. operant chamber, open field, home cage, etc.) require customized separation criteria	[195]
Automatic mouse ultrasound detector (AMUD)	An algorithm for the automatic detection and extraction of USV syllables which runs on STx acoustic software The de-noising steps are amplitude-sensitive Provides information on the detected element frequency, amplitude, and time variables For detecting USV that are not shorter than 10 ms	[196]
Vocal inventory clustering engine (VoICE)	A classification software that utilizes acoustic similarity relationships between vocal events to generate high dimensional similarity matrices, which are then subjected to hierarchical clustering based on mean frequency and each note's slope, duration, and curvature Based on pre-defined rules, the syllables are clustered into a limited number [9–12] of named categories Includes syntactical similarity quantification to detect changes in syllable patterns across conditions Independent method is needed to detect and ‘‘clip’’ each syllable into a separate wav file	[190]
Mouse song analyzer (MSA)	A custom MATLAB program based [163] modified from code written by [167] and further developed by [164] for automated, rule-based categorization of syllable shapes Multi-note syllables are classified based on the number and direction of frequency jumps (or pitch jumps) but not based on the duration, slope, or curvature of each note The detected syllables are categorized into a limited number [4–15] of named categories based on pre-defined rules Other measured variables include syllable duration, inter-syllable interval, standard deviation of pitch distribution, pitch mean frequency, frequency modulation, and spectral purity [164] Offers syntax composition and probability analysis to determine the probability of transitioning between different syllable types within a given context, an analysis that enables the identification of repeated syllable patterns (e.g., songs)	[163, 164, 167]
Mouse Ultrasonic Profile ExTraction (MUPET)	An open access MATLAB tool for data-driven analysis of USVs by measuring, learning, and comparing syllable types MUPET uses an automated and unsupervised algorithmic approach for the detection and clustering of syllable types summarized in the following features: Syllable detection by isolating and measuring spectro-temporal syllable variables, followed by analyzing overall vocalization features (syllable number, rate and duration, spectral density, and fundamental frequency) The application of unsupervised machine learning based on k-means clustering to build “syllable repertoire” from the dataset which includes up to several hundreds of the most represented syllable types based on spectral shape similarities within that dataset Similarity measurement between syllable types of two different repertoires using rank order comparisons in a manner that is frequency-independent Centroid-based (k-medoids) cluster analysis of syllable types from different syllable repertoires of different datasets to measure the frequency of use of different syllable types across conditions or strains and identify shared and unique shapes Provides automated time-stamps of syllable events for synchronized analysis with behavior The option for the user to control features regarding noise reduction, minimum and maximum syllable duration, minimum total and peak syllable energy, and the minimum inter-syllable interval needed to separate rapidly successive notes into distinct syllables Cannot detect USVs below 30 kHz [197]	[166]
DeepSqueak	A USV detection and analysis software suite based on regional convolutional neural network architecture to detect and categorize USV calls syllables Packaged with four default detection networks: one general-purpose network, one for mouse USVs, one for short rat USVs and one for long 22 kHz rat USVs Detecting USVs is done by a region proposal network, which segments the filtered sonogram image into proposed areas of interest with possible USVs, which are then passed to the classification network to determine whether the image contains a call or background noise. The detected USVs are then saved to a detection file along with call variables and classification confidence scores An option for creating and training custom de-noising secondary networks (by manual annotation of noise vs. call) for identifying noises that might be specific to certain experiments\setups For syllable clustering, the user can determine which USV features are most important for clustering and adjust three weighted input features that are contour-based: shape, frequency, and duration (thus clustering is amplitude invariant). The number of clusters can be determined by the user using supervised neural networks, or by unsupervised data-based clustering by using k-means on perceptually relevant dimensions of the extracted contour to place calls into a predefined number of clusters	[197]
USVSEG	A program for detecting USV segments (syllables) in continuous sound data containing background noise from several rodent species Output contains segmented sound files, image files, and spectral peak feature data that can be used for clustering, classification, or behavioral assessment using other toolkits	[198]
VocalMat	A software that uses image-processing and differential geometry approaches to detect USVs in spectrograms, thus eliminating the need for user-defined parameters or costume training of the neural network VocalMat uses computational vision and machine learning by training a convolutional neural network to classify detected USVs into distinct 11 USV categories or noise	[199]

While mice USVs do not clearly depict the emotional state of the emitter [168] as in rats, they still serve a communicative function that modulates interactions in social contexts among both males and females [158]. USVs of male mice were mainly investigated in the context of reproduction [175]. When exposed to a female, male mice emit USV songs composed of different types of syllables repeated in regular, temporal, and non-random sequences [167] (Fig. 3D, E). Although USV emission in mice is innate [176], it is highly influenced by social experience. For example, the acoustic features and syllable variables of male USVs to females are affected by the receptivity of the female (specifically, its estrous state) [169], the state of the female (i.e., vivid, anesthetized, or urine only) [164], female presence [169], and prior sexual [177] and social experience [161, 165, 178]. Male courtship USVs were found to be mediated by a distinct neural population in the PAG connected to downstream premotor vocal-respiratory neurons in the nucleus retroambiguus to control the temporal and spectral features of the emitted USVs [179]. In a playback study, female mice were also shown to favor male songs over pup vocalizations and noise [180]. Together, these findings demonstrate that the USVs of adult male mice facilitate the attraction of females and promote reproduction. However, adult male mice were found to emit USVs in other social contexts with same-sex stimuli (Fig. 3 F–H), namely, in response to a male intruder [175], and during interactions with a male stimulus following social isolation [165, 170, 174]. Males also emit low-frequency USVs (≤ 60 kHz) when held in a constrainer with a nearby male conspecific [174], indicating that USVs in adult male mice serve a broader social function than merely courtship calls.

As for female mice, earlier studies showed that interactions between devocalized males and intact females abolished detected USVs, while interactions between intact males and devocalized females had little effect on the number of detected USVs, thus leading to the conclusion that USVs in male–female interactions are mainly emitted by the male [181]. However, later research found that female mice do vocalize during interactions with males, although to a lesser extent of 15–18% of the total USVs recorded [159, 160, 182, 183] and with USVs of different acoustic features than those of males [159, 162, 183]. Females also emit USVs in female-female interactions [184, 185] and in response to an awake or anesthetized female intruder in the resident-intruder test [165, 186] (Fig. 3I–K). USVs of females in same-sex interactions are influenced by their motivational state and sexual receptivity, age, familiarity [187], and prior social isolation [161, 165]. Therefore, USVs in females appear to serve many roles, including territorial calls [186], indexing familiarity [187], and facilitating approach [165, 185].

It should be pointed out, however, that some experimental designs include isolating subjects prior to the experiment so as to induce emission of a greater number of USVs [159, 167, 184, 187], which may compromise the generality of the results by introducing the confounding effects of isolation on USV emission and social behavior in both males [162, 170, 174] and females [165]. To overcome such limitations, de Chaumont et al. [37] recorded same-sex pairs of mice over three days in a home-like environment to examine differences in spontaneously emitted USVs without the contribution of prior isolation or constrained interaction in limited recording sessions. This method uncovered changes in USVs that were dependent on age, sex, genotype, and social context, signifying a possible role for USVs as an indicator of higher arousal states in social interactions.

**Fig. 4:**
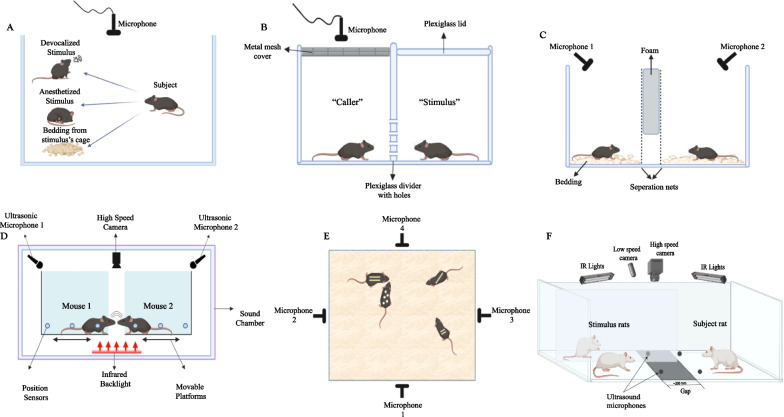
Experimental set-ups for identifying a vocalizing subject. **A** Identifying a vocalizing subject by neutralizing vocalizations of a stimulus by any of various means, including devocalization, anesthesia, or presenting the subject with bedding from the stimulus’s cage, **B** placing the stimulus in a plexiglass-covered compartment for recording USVs from the subject’s compartment alone, **C** using two microphones positioned at opposite ends above the compartments of the interacting animals in a divided arena, **D** using two microphones positioned at opposite ends of an arena in which nose-to-nose-interacting subjects are placed on separate platforms, **E** using a four-microphone array for recording vocalizations from multiple animals during free interaction, and **F** using four microphones while recording from two interacting animals across a gap

The research of ultrasonic vocalizations is currently hindered by technical challenges that include determining the identity of the vocalizer during interactions with two or more animals. Both males and females can emit USVs of similar features [182, 186], and mice do not show clear visual cues of their vocal behavior [183]. Attempts to overcome this challenge included surgical interventions to devocalize one of the interacting animals, specifically by unilateral incision of the inferior laryngeal nerve [181, 188], anesthetizing the stimulus [170, 172, 186], or exposing the subject to urine or bedding collected from the stimulus instead of a conspecific [167, 177]. To attain vocalizer identity without any outside intervention, Zala and colleagues [160] recorded USVs from subjects interacting with a stimulus through a plexiglass divider wall, with the compartment of the stimulus being covered in a plexiglass lid to ensure that only USVs from the subject’s compartment were recorded. While promising, this method entails the placement of a separator between the subject and the stimulus, thus limiting the extent of social interaction. In contrast, Neunuebel et al. [182] used a four-channel ultrasonic microphone array-based system combined with a sound source localization method for localizing the source of the recorded USVs in groups of freely-behaving mice. By using four microphones, multiple estimates for a given sound signal were extracted and then averaged to pinpoint the location of the source. Combined with video tracking of mice location, a probability index for each mouse was then calculated to assign the source of the sound. While this system allows for analysis of USVs in freely behaving animals, it is not without limitations, as the median error between the location of the actual mouse and the estimated sound source is 3.87 cm, with an identity localization percentage of 78.03% of the total detected USVs. Heckman et al. [183] recorded USVs of two nose-to-nose-interacting mice placed on two separate platforms using two microphones arranged at either side of the arena for an accurate estimation of the vocalizing mouse based on temporal differences in sound time arrival (Fig. 4). A set-up with a similar premise was used in Rao et al. [189] to investigate how the interplay between facial touch and USVs modulates the activity of the auditory cortex. The set-up included a gap between the platforms of the subject and stimulus rats, allowing for only close face-to-face interactions. USVs were recorded with four ultrasonic microphones, such that the identity of the caller was assigned by intensity measurements to yield a success rate of 80% of the detected USVs. Notably, both set-ups described in Heckman et al. [183] and Rao et al. [189] reduced the social interactions under investigation to only one dimension, thus limiting the extent of physical contact between the examined subjects, which might in turn have limited the repertoire of USVs emitted. On a related note, our lab is currently developing mini-microphones directly implanted into a subject’s head for accurate recognition of the emitter’s identity and a more sensitive detection of a broader range of USVs than is usually detected by a microphones placed above the arena.

Given their communicative function in social interactions, ultrasonic vocalizations seem to be worthy candidates for a translational endophenotype in studying socio-emotional interactions and modeling neurodevelopmental disorders with deficits in communication, such as ASD. Indeed, abnormal emission of USVs among pups and adult subjects has been reported in multiple genetic models of ASD, including *16p11.2* [39, 84], *Cntnap2* [38, 83, 190], *Iqsec2* [73], *Shank2* mice [191], and *Shank3* mice [36, 37, 55, 192, 193] and rats [28], as well as BTBR mice [36, 175, 177, 194], emphasizing the translational and face validity of USVs as a model for socio-affective communication. Therefore, the analysis of USVs in terms of frequency, sequence, number, and acoustic structure may provide greater insight into the state of the tested animal and how these are altered under various manipulations and abnormal conditions, as well as offer a potential platform for evaluating the efficacy of therapeutic assays in the context of ASD.

### Urinary scent-marking

Rodents use urinary scent marks (among other means) for communicating with conspecifics in many social contexts, including individual recognition, assertion of dominance, and reproductive status assessment [36, 49, 109]. Urinary scents convey information regarding the sender's age, sex, strain, social status, health fitness, and individual identity [108]. Communication through urinary scents is mediated mainly by two classes of protein, specifically the major histocompatibility complex (MHC) and major urinary proteins (MUPs) [108, 200]. Scent marking through urine changes over the course of development, with increased urination by C57BL/6J mice being seen after exposure to a novel CD1 stimulus or a female stimulus appearing only after sexual maturation at the age of 2–3 months [200]. In male-to-male interactions, urinary marking is used for territorial establishment, and is influenced by dominance and suppressed by social defeat [177]. It also influences inter-male aggression and the display of attack behavior, an observation mediated by neural projections from the vomeronasal organ (VNO) via the bed nucleus of the stria terminalis (BNST) to dopaminergic neurons in the ventral pre-mammillary nucleus of the hypothalamus (PMv) [201]. Territorial urinary marking in the presence of urine from a novel conspecific was found to be influenced by social experience and enhanced by prior social isolation, an effect mediated by the activity of the lateral hypothalamus [202]. Urinary marking also conveys social memory and habituation to a given conspecific, since repeated exposure to the same conspecific is correlated with a reduction in urinary scent-marking in C57BL/6J male mice and is recovered upon the introduction of a novel stimulus [108]. Urinary scent detection also influences the arousal state and behavior of the receiver. For example, non-lactating female mice exposed to the major urinary protein darcin present in male urine emitted a greater number of USVs and showed an increase in scent-marking behavior for communicating reproductive status, a behavioral effect mediated by the medial amygdala [109]. Interestingly, deficits in urinary marking by male subjects in response to urine from a female in estrus were found among BTBR [177] and *Cntnap2*^−\−^ but not among *16p11.2*^df\+^ mice [83], indicating that urinary scent-marking is disrupted in some models of ASD but not in others.

Some of the methods presently used for tracking urination in mice rely on post hoc analysis of urine spots collected by placing absorbent paper underneath the subjects. Urine spots can then be detected and analyzed by fluorescence imaging, given that urine presents red-shifted fluorescent emission when excited with UV light [202–204], or by using Ninhydrin spray for urine fixation [108, 200, 205]. Such methods can only provide information on the cumulative output of voiding behavior, like void numbers, volume, and spatial distribution. However, these methods are incapable of detecting the exact time of each void and differentiating between two overlapping voids, and are poorly suited for combined analysis of other time-sensitive methods, like brain activity [206, 207]. In contrast, thermal imaging offers a promising solution to such limitations, due to the ability to detect voiding events on the basis of the distinctive thermal signature of urine, namely how freshly deposited urine is close to body temperature and then cools down below ambient substrate temperature. Thermal imaging thus offers a valuable and highly informative tool for investigating the spatial and temporal dynamics of micturition behavior in social contexts that can be combined with other in vivo methods for uncovering possible interplay with other sensory cues or unmasking of the neural processing mechanisms underlying such behavior. For example, using thermal imaging, Miller et al. [206] were able to investigate the spatiotemporal dynamics of micturition in male mice and uncover changes in scent-marking signaling behavior in response to different social contexts and the outcome of prior social competition that could not have been detected by other post hoc methods for urine spot analysis.

Taken together, these findings indicate that scent-marking represents an active emission of a signal that serves a social function between conspecifics with the ability to convey and induce changes in the socio-affective behavior in various contexts, and thus merits further investigation.

### Sniffing

Sniffing is an active respiratory behavior essential for acquiring and sampling odors typically exhibited at a higher frequency than average respiration rates and commonly displayed during motivated behaviors, such as social behaviors [139, 154]. Highly aggressive rats were shown to display decreased sniffing during exploration of a novel context accompanied by increased anxiety-related behaviors, indicating that sniffing can be used as a physiological marker for measuring the arousal state of an animal [208]. In addition, sniffs and other orofacial behaviors, like whisking and changes in head position, show oscillatory patterning at theta frequency (4–12 Hz), a frequency that reflects arousal and is relevant to information exchange between brain areas [154, 209]. Abnormalities in sniffing were also correlated with reduced social [38] and sexual [39] drive in mice in which the *Cntnap2* gene was knocked-down in the PFC and in *16p11.2*^+/−^ mice, respectively. Practically, sniffing can be monitored by connecting a cannula implanted into the nasal cavity of the animal to a pressure sensor for monitoring airflow [154, 209], allowing the detection of sniffing patterns at the millisecond time resolution and providing the ability to integrate other methods of analysis to the set-up. Therefore, research of neural correlates in social behavior will benefit from examining sniffing patterns and their changes during specific events in social interactions, as well by examining their relationships with other communication modalities.

### Facial expressions

In humans, facial expressions offer a generous source of information for conveying the subjective emotional experience and for inferring the emotional experience of others [100]. In rodents, however, the importance of facial expressions as a modality for inter-species communication might be less pronounced than it is in humans, given that rodents are olfactory creatures with weakly developed facial musculature and relatively poor changes in their facial expressions [54]. Still, recent evidence suggests that facial expressions in rodents convey information regarding the individual's emotional state. Mice, for example, display distinct changes in their facial expressions, including bulges in the nose and cheeks, and changes in the positions of their ears and whiskers in response to noxious stimuli that were utilized to establish a mouse grimace scale for assessing pain response in mice [210]. Mice also show tightened eyes and flattened ears in response to an intruder in an resident-intruder test but not in response to a cat odor [211]. Distinct facial expressions were also detected in positive contexts among rats who showed significant ear color and ear angle changes during tickling [212]. Recent and highly compelling evidence for the display of distinct facial expressions in mice was provided by Dolensek et al. [110], who generated a non-supervised algorithm to cluster and classify facial expressions. In this manner, distinct facial expressions mediated by “face-responsive” neurons in the insular cortex were detected and correlated with different emotional events, including disgust, pleasure, malaise, active, and passive fear.

While the communicative value of facial expressions and their importance in directing the behavior of the observer in rodents is still unclear, the analysis of facial expressions can still provide unique insight into the affective state experienced by an animal and thus can be used to assess affective responses to certain stimuli/treatments and how these are altered under pathological conditions.

### The behavior of the stimulus

Social interactions in nature are rarely unilateral and often entail instantaneous changes in behavior and mutual feedback between multiple participants. Still, behavioral tasks used for estimating social behavior usually restrict the physical expanse of the social interaction and focus on the behavior of an individual subject, neglecting the dyadic nature of social interactions and the contribution of the stimulus in driving the behavior of the subject. Vocalizations emitted by the stimulus can influence the behavior of the subject and vice versa [152, 153]. Rats, for instance, showed preference and induced approach to 50 kHz calls in playback studies [152], demonstrating the ability of the emitter to influence the behavior of the receiver. Interestingly, this pro-social effect of 50 kHz calls was found to be absent in male but not female Shank3^−\−^ rats, indicating reduced social motivation [28]. Rats also display cognitive bias in an Ambiguous-Cue Interpretation Test induced upon hearing USVs of certain frequencies. Rats exposed to 50 kHz USVs showed optimistic bias in the judgment of an ambiguous tone, while rats exposed to 22 kHz calls showed a pessimistic bias, indicating that USVs are capable of influencing the emotional state of the listener [213]. Mice were also shown to exhibit elevated corticosterone levels and anxiety-related behaviors when listening to mid-frequency calls in playback [106], further confirming that vocalizations emitted by the stimulus can alter the affective state of the subject. Also, mice exposed to multiple stimuli held in enclosures that allow varying levels of sensory cues to be detected by the subject showed an increased probability of investigating stimuli held in the enclosure permitting the highest level of social cues complexity [3]. In addition, mice exposed to an anesthetized intruder in a resident intruder test emitted USVs with different acoustic structures in terms of duration and number of frequency jumps than those emitted when the subjects were introduced to an awake intruder, demonstrating that USVs emitted by a subject are influenced by the state of the stimulus and the arousal level induced by the stimulus [186]. These results indicate that the integration of multiple sensory cues emitted by a stimulus is important for a more salient representation of the stimulus and for driving the social behavior of a subject [3]. Another cue that can influence the behavior of a subject is a stimulus’s movement. Stimuli differing in familiarity to a subject (i.e., a familiar cage-mate versus a novel mouse) were shown to exhibit a different number of large movements as measured by piezoelectric sensors, which in turn had a differential effect on the social investigation of the stimulus by the subject [70]. Therefore, while it is important to capture changes in the behavior of a subject in response to a given social stimulus, examination of a stimulus’s behavior and affective state can provide further information on the exact nature of the influencing variables driving the observed changes in the behavior/neural activity of the subject. Overall, the contribution of a stimulus’s behavior and affective state should also be included when analyzing any social interaction.

### Interaction between multimodal cues

**Fig. 5:**
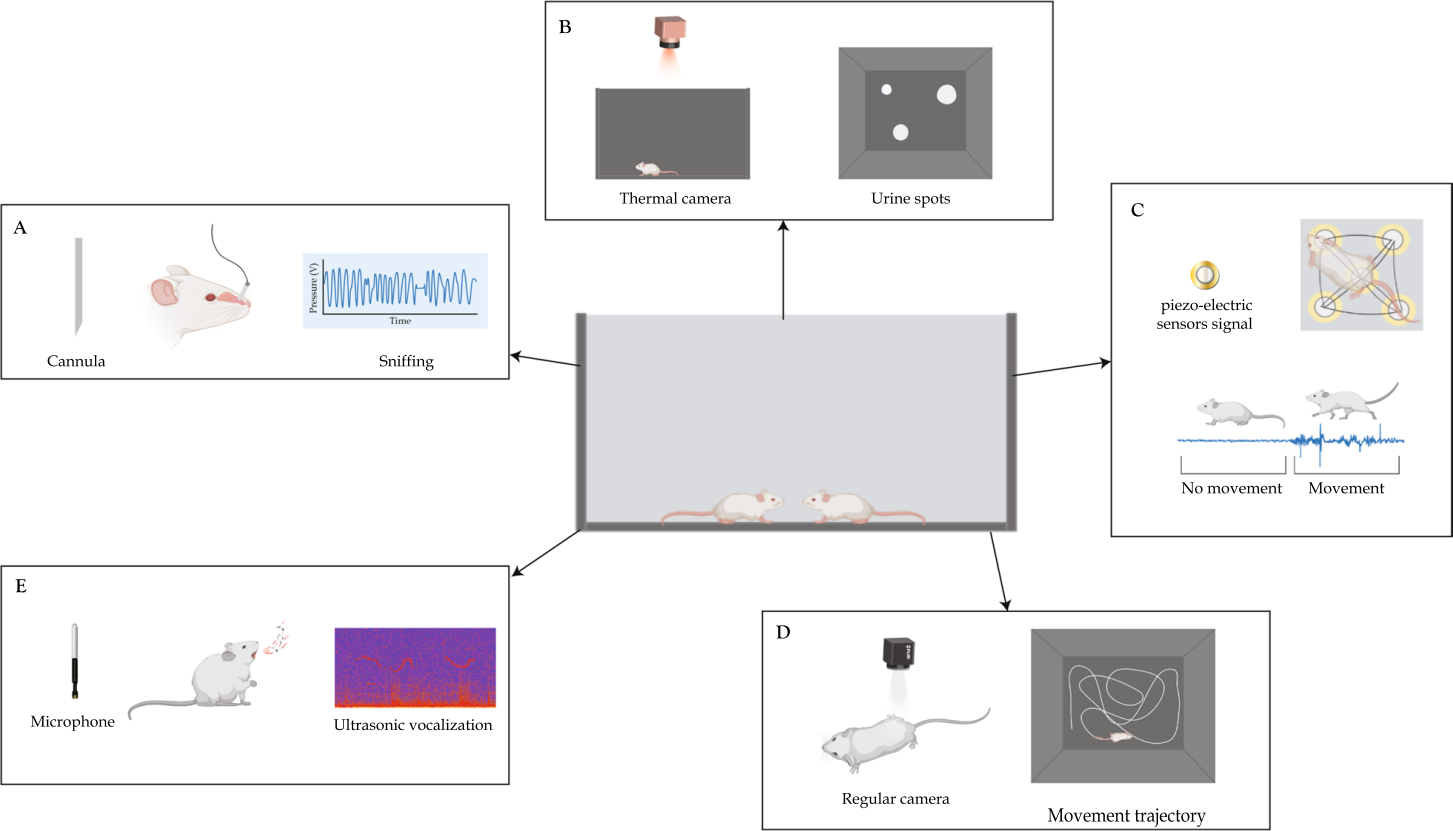
A multimodal approach for social behavior analysis. **A** Cannula implantation into the nasal cavity for measuring sniffing patterns. **B** Imaging with a thermal camera that captures micturition and urinary scent-marking. **C** Piezo-electric sensor array for capturing animal movements. **D** Imaging with regular cameras for overall analysis of behavior. **E** Microphones for capturing ultrasonic vocalizations

Whereas isolating and focusing on one variable is important for detailed understanding of its role and influence, it is also important to keep in mind that focusing on one pixel does not convey the whole picture. Indeed, modalities of social communication rarely work in isolation and are often simultaneously synchronized with other modalities. One such example is sniffing. Wesson [139] showed that changes in sniffing behavior communicate social hierarchy and influence the latency to be attacked by a dominant subject. However, later work by Sirotin et al. [154] showed that active sniffing and ultrasonic vocalizations during social interactions bidirectionally influence one another, with USVs being strictly emitted during periods of active sniffing, especially in the exhalation phase, thereby altering the sniffing phase which modulates segmentation of ultrasound production into individual calls. Such findings suggest that alterations in sniffing can be caused by or coupled with the emission of USVs. Later, Alves et al. [209] showed that changes in sniffing are correlated with other orofacial behaviors, like head movements in the x- and y-axis in a manner influenced by the walking speed of the animal. Therefore, a better and more inclusive understanding of a subject’s emotional state requires not only strict analysis of a given variable during social behavior but also examining its interplay and correlation with other variables (Fig. 5).

## Conclusions

Establishing the existence of social deficits in mouse models for any given disorder requires reliance on more than one behavioral paradigm or variable, given that such an approach is binary, context-dependent, and susceptible to pollution by various confounding variables, which could compromise the conclusiveness of any findings. Therefore, phenotyping behavioral deficits in animal models should implement the systematic use of a battery of behavioral tasks that address different aspects and contexts of social behavior. In addition, incorporating various methods for detailed and automatic analysis of multiple physiological and behavioral variables during tasks and combining these variables with brain recordings and machine-learning algorithms will allow for determining and characterizing socio-emotional states during social interactions of animal subjects who encounter various types of social stimuli. Such an integrative approach for analyzing social behavior in rodents not only will accelerate investigation of the brain mechanisms involved, but will also enable a genuine comparison of deficits between human patients and animal models of pathological conditions.

## Data Availability

Data sharing is not applicable to this article as no datasets were generated or analyzed during the current study.
